# Supplementary material for: Foveal Hypoplasia in CRB1-Related Retinopathies
Source: Int J Mol Sci. 2023 Sep 11;24(18):13932. doi: 10.3390/ijms241813932 (PMC10531165; doi:10.3390/ijms241813932)
Supplement: Supplementary file 1 [file ijms-24-13932-s001.zip › ijms-2550484-supplementary.pdf]

Supplemental Material

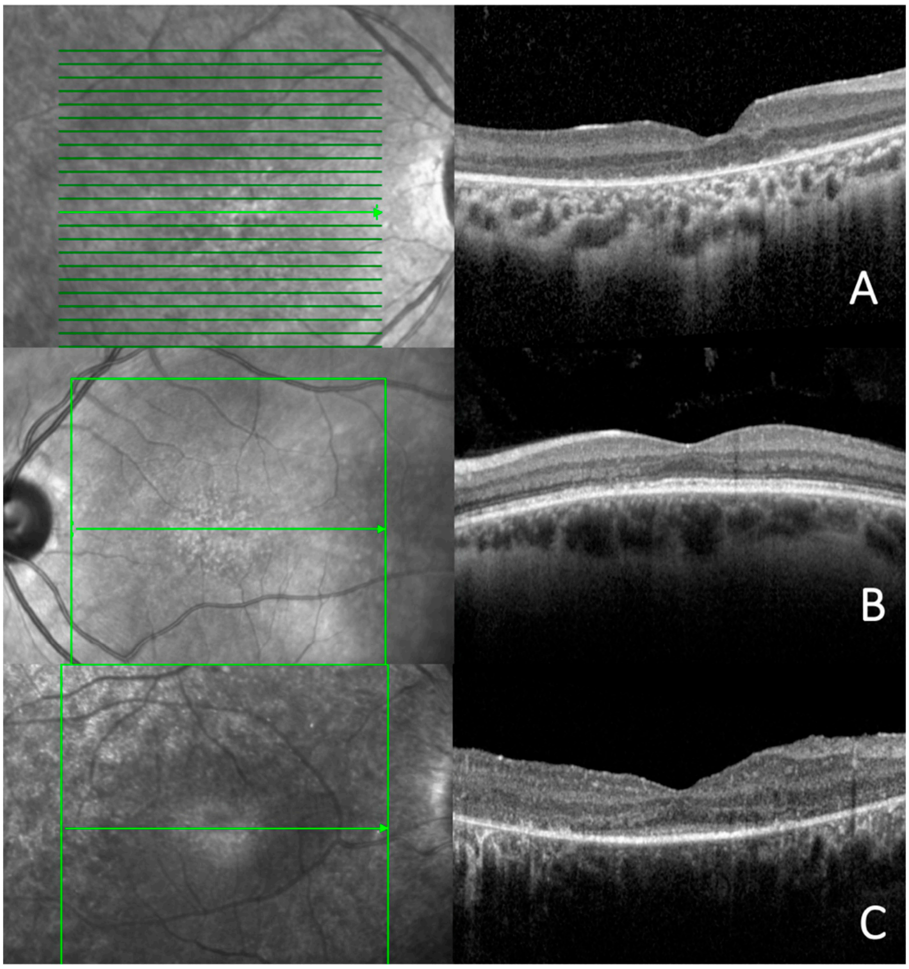

Supplemental Figure S1. Multimodal imaging in representative patients with FH. (**A**) Grade 1a FH (**B**) Grade 1b FH **(C)** Grade 1b FH.
